# Cellulose nanofiber-reinforced solid polymer electrolytes with high ionic conductivity for lithium batteries†

**DOI:** 10.1039/d3ta00380a

**Published:** 2023-04-04

**Authors:** Cristina Prado-Martínez, Preston Sutton, Isabella Mombrini, Aristotelis Kamtsikakis, Worarin Meesorn, Christoph Weder, Ullrich Steiner, Ilja Gunkel

**Affiliations:** a Adolphe Merkle Institute, University of Fribourg Fribourg 1700 Switzerland ilja.gunkel@unifr.ch; b ARC Centre of Excellence for Electromaterials Science and Institute for Frontier Materials, Deakin University Melbourne 3125 Australia; c Electrochemical Innovation Lab, UCL London UK

## Abstract

Lithium–metal electrodes are promising for developing next-generation lithium-based batteries with high energy densities. However, their implementation is severely limited by dendritic growth during battery cycling, which eventually short-circuits the battery. Replacing conventional liquid electrolytes with solid polymer electrolytes (SPEs) can suppress dendritic growth. Unfortunately, in SPEs the high stiffness required for suppressing dendrites comes at the expense of efficient lithium-ion transport. Some polymer-based composite electrolytes, however, enable the decoupling of stiffness and ionic conductivity. This study introduces a composite SPE comprised of a relatively soft poly(ethylene oxide-*co*-epichlorohydrin) (EO-*co*-EPI) statistical copolymer with high ionic conductivity and cellulose nanofibers (CNFs), a filler with extraordinary stiffness sourced from abundant cellulose. CNF-reinforcement of EO-*co*-EPI increases the storage modulus up to three orders of magnitude while essentially maintaining the SPE's high ionic conductivity. The composite SPE exhibits good cycling ability and electrochemical stability, demonstrating its utility in lithium metal batteries.

## Abstract 2

The composite solid polymer electrolyte (SPE), a soft copolymer reinforced with cellulose nanofibers, provides both high ionic conductivity and stiffness to suppress dendrite growth, thereby enabling high-energy-density lithium metal batteries.

## Graphical abstract

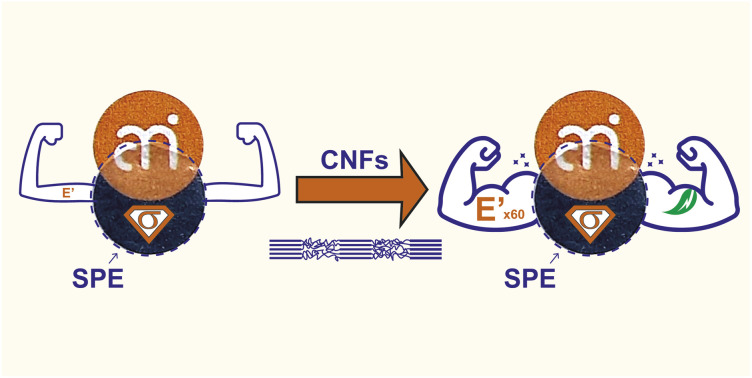

## 1 Introduction

The design of next-generation batteries often involves Li metal as an anode as this would substantially improve the cell's energy density compared to currently employed graphite anodes.^1–3^ However, the non-uniform plating of Li metal prevents its practical use as an anode. The repeated stripping and redeposition of Li during cycling results in Li dendrites, which can bridge the anode–cathode gap causing internal short circuits.^4^

A potential solution to this problem commonly put forward is the increase of the elastic modulus of the electrolyte.^5–8^ A sufficiently high work required for the deformation of this solid material should suppress the above-described electrode instability. This can be achieved, for example, with stiff solid polymer electrolytes (SPEs). Apart from their promise to enable Li metal anodes, SPEs address other limitations of liquid electrolytes by their relative non-flammability, easy processability, flexibility, and electrochemical stability.^9^ Numerous SPE systems have been developed in the past decades, but the most intensively studied materials are poly(ethylene oxide) (PEO)-based electrolytes due to their good lithium ion-solvation and ionic transport properties.^8,10–12^

However, there is a conundrum when using PEO and other SPEs in combination with Li-metal anodes. Suppressing dendritic Li metal growth requires a high elastic modulus, which for polymers implies a low mobility of their segments. At the same time, lithium ion transport in polymers is coupled to segmental motion, *i.e.* high lithium ion mobility requires a high mobility of the polymer segments. Thus, the ongoing challenge in developing SPEs is formulating materials that simultaneously display a high elastic modulus and high Li-ion mobility, two seemingly contradictory requirements. The main drawback of PEO stems from its semi-crystalline morphology at room temperature since only the amorphous portion contributes to bulk ionic conductivity.^13^ Heating the electrolyte above its melting point (*ca.* 70 °C) increases the conductivity sufficiently for practical use. However, crystal melting also reduces the mechanical strength, compromising the electrolyte's ability to inhibit short circuits due to Li dendrite growth.

While simple polymer electrolytes, such as lithium salt-doped PEO, are unsuitable for addressing the above challenges, they can play an important role in composite electrolytes. For example, the addition of organic and inorganic plasticisers^14^ increases the conductivity of PEO-based electrolytes due to enhanced polymer chain mobility and reduced crystallinity. Their mechanical strength can be increased by cross-linking,^9^ copolymerisation with glassy blocks,^15^ or by adding reinforcing agents.^16^

Reinforcing SPEs with rigid fillers allows for particularly facile tuning of conductivity and stiffness as the filler content in the composite SPE can be readily adjusted. Nanocellulose-based fillers are of particular interest as they provide effective reinforcement while being renewable.^17–19^ Cellulose is the most abundant biopolymer on earth and can be easily extracted from numerous natural sources, particularly agricultural waste products. It can be processed into various forms, such as cellulose nanocrystals or cellulose nanofibers (CNFs), which both show high stiffness but differ in their aspect ratio and crystallinity.^17^ The reinforcing effect of such cellulose-based reinforcing agents has been demonstrated for various SPEs. Chinnam *et al.* synthesised a blend of LiClO_4_, methylcellulose (MC), and oligomeric dendritic polyethylene glycol (PEG).^20^ Increasing the ratio of MC in the blend also increased the elastic modulus up to 109 MPa, albeit at the expense of Li-ion conductivity, which reached a maximum of 1.5 × 10^−4^ S cm^−1^ at 90 °C. Chen and coworkers^21,22^ explored cellulose non-woven reinforcement with two types of polymer blends, obtaining SPEs that performed well over 50 cycles^21^ and 500 cycles.^22^ The Young's moduli of these blends were 25 and 43 MPa, respectively, demonstrating the efficacy of SPEs with relatively low elastic modulus in the suppression of dendrite formation. Dufresne and coworkers also used cellulose nanocrystals (CNCs) in various studies^23–27^ to reinforce PEO. While the stiffness was increased, the Li-ion conductivity typically decreased with increasing CNC content, except when a cross-linked polyether SPE was employed,^23^ in which the Li-ion conductivity was only marginally affected by the addition of the filler. A study with different types of CNCs with different aspect ratios^24^ exhibited a positive correlation between the aspect ratio of the filler and the elastic modulus. More recently, other cellulose-based fillers were used to reinforce PEO, resulting in composite SPEs for Li batteries with promising performance, including high stiffness and high ionic conductivity.^28,29^

Cellulose nanofibers (CNFs) are interesting for practical battery designs. CNFs are typically several micrometres long and several nanometres wide, *i.e.*, they have a very high aspect ratio. When added in sufficiently high concentration, CNFs form a reinforcing network in polymeric matrices. Unlike CNCs, which generally show high crystallinity, CNFs contain both amorphous and crystalline domains,^30^ and despite their high elastic modulus of up to 140 GPa for single fibres,^31^ their length renders them quite flexible, which provides high flexibility and results in the formation of entanglements, which is beneficial for reinforcing the composite and in the suppression of crack propagation.^32^ CNFs were used to reinforce a range of different polymers, with substantial increases in the elastic modulus.^32–35^ CNFs were also used to reinforce polymer electrolyte gels for Li-ion batteries, but this was achieved at the expense of Li-ion conductivity, which decreased by more than one order of magnitude.^36,37^ However, as recently demonstrated for solid polymer electrolytes using PEO as a matrix, CNF reinforcement remains promising for the design of SPEs for lithium batteries.^38^

Poly(ethylene oxide-*co*-epichlorohydrin) (EO-*co*-EPI) statistical copolymers are interesting for the use as SPEs. In these copolymers, the crystallinity decreases with the content of EPI. An optimized composition with a molar EO : EPI ratio of 84 : 16 doped with 5.5% (w/w) of LiClO_4_ showed an ionic conductivity of 4.1 × 10^−5^ S cm^−1^ at 30 °C.^39^ The improvement of the mechanical strength and stiffness of this material was the motivation of subsequent work on the CNC reinforcement of EO-*co*-EPI copolymers SPEs.^40^ While the elastic modulus increased by more than a factor of 50 with a low loss in Li-ion conductivity, a relative humidity of 75% was, however, required to achieve fair ion conductivity values, which is impractical for Li batteries. Thus, a dry EO-*co*-EPI copolymer-based composite that is compatible with Li metal anodes remains to be demonstrated.

Here, we describe a dry solid polymer electrolyte nanocomposite containing an EO-*co*-EPI copolymer, lithium trifluoromethane-sulfonyl imide (LiTFSI), and cellulose nanofibers. The CNFs endow the material with sufficiently high stiffness to fabricate self-standing films. When employed as SPE in cells with Li-metal anodes, these new composite electrolytes resist dendrite formation while maintaining good ionic conductivity. Its statistical copolymer matrix also extends composite electrolytes beyond the common linear PEO homopolymer matrix.

## 2 Experimental section

### 2.1 Materials

The statistical poly(ethylene oxide-*co*-epichlorohydrin) (EO-*co*-EPI) copolymer with a molar EO : EPI ratio of 84 : 16 (*M*_n_ = 320 kg mol^−1^, *M*_w_ = 1380 kg mol^−1^) was provided by Osaka Soda Co. and used as received. Tetrahydrofuran 99.8% (THF) and bis(trifluoromethane)sulfonimide lithium salt (LiTFSI) were purchased from Sigma-Aldrich and were used without further purification.

Cellulose nanofibers (CNFs) having an average width of 29 ± 11 nm were provided by Dr Gilberto Siqueira (EMPA, Switzerland) in the form of an aqueous slurry with a CNF concentration of 15 mg g^−1^, as determined gravimetrically. To disperse the CNFs in THF, the aqueous slurry was mixed with this solvent, and the mixture was centrifuged three times at 8000 rpm for 15 min in a Jouan B4 centrifuge. After each cycle, the supernatant was decanted and replaced with 30 g of fresh THF. The final CNF/THF suspension with a concentration of 2.5 mg CNFs/g THF was stirred for 1 h at room temperature.

### 2.2 Preparation of the SPE

EO-*co*-EPI/CNF/LiTFSI composites containing 10% w/w of CNFs and various LiTFSI/EO ratios were prepared by solution-casting from THF. The CNF/THF suspension was added to a 5% w/w EO-*co*-EPI THF solution that had been previously prepared and the mixture was stirred for 30 min. A LiTFSI/THF solution (200 mg LiTFSI/g solution) was added to achieve the desired molar Li/EO ratio, and the mixture was sonicated in a Sonoswiss SW3H ultrasonic bath for 2 h before casting it into a round Teflon Petri dish (10 cm diameter), followed by drying at 50 °C overnight. The dried films were compression-moulded in a Carver press between Teflon sheets at 70 °C for 5 min at a pressure of 1500 psi resulting in whitish translucent films with a thickness of *ca.* 100 μm. The samples were cut and then dried under N_2_ atmosphere in a vacuum oven at 70 °C overnight and then transferred into a glovebox. In all samples, *r* denotes the molar ratio of Li^+^ ions to ether oxygens in the EO segments, as ether oxygens in the EPI are not involved in the ionic conductivity.^39^

### 2.3 Transmission electron microscopy (TEM)

As previously described,^41^ transmission electron microscopy (TEM) images of the CNFs were acquired to determine the dimensions of the nanofibers. Aqueous dispersions of CNFs (0.03% w/w) were spin-coated (4 μl, 3000 rpm, 1 min) onto plasma-treated formvar/carbon TEM grids. The samples were imaged without staining using a transmission electron microscope (FEI Tecnai Spirit, US) at a voltage of 120 kV. Size analysis was conducted using ImageJ's image processing software from five TEM images (sample size *n* = 150).

### 2.4 Fourier-transform infrared spectroscopy (FTIR)

Spectra were recorded on a PerkinElmer Spectrum 65 spectrometer in the range of 4000 cm^−1^ to 550 cm^−1^ with a resolution of 4 cm^−1^ and an average of 8 scans per sample. The SPEs were dried in a Binder vacuum oven (BINDER GmbH, Tuttlingen, Germany) at 70 °C for approximately 24 h prior to characterization. The precursor materials were measured without any drying.

### 2.5 Differential scanning calorimetry (DSC)

DSC measurements and analysis were performed with a Mettler-Toledo STAR under a N_2_ atmosphere. Samples of approximately 10 mg were placed in a 40 μL Al pan and cycled twice between −80 °C and 130 °C at 10 °C min^−1^. The DSC curves shown correspond to the second heating.

### 2.6 Dynamic mechanical analysis (DMA)

The mechanical properties of the SPE were studied with a dynamic mechanical analyser (DMA, TA instruments Model Q800) in tensile mode. The tests were conducted at a frequency of 1 Hz and a strain amplitude of 15 μm in the temperature range of −70 °C to 150 °C at a heating rate of 5 °C min^−1^. Rectangular films with a length of approximately 10 mm, a width of 5.17 mm and thicknesses of *ca.* 100 μm and 300 μm were used. The mechanical data shown in Fig. 2 and S5† represent average values of 3–5 individual measurements.

### 2.7 Electrochemical impedance spectroscopy (EIS)

Conductivity measurements of the SPE between room temperature and 95 °C were carried out by electrochemical impedance measurements using a BioLogic SP-300. The SPE was sandwiched between two polished stainless steel disks with replaceable springs inside a Swagelok cell. The temperature was controlled in a Binder oven with an approximately 1 h dwell time for temperature equilibration. Typical measurements were taken in potentiostatic mode with a 10 mV AC potential applied at frequencies ranging from 1 Hz to 1 MHz. Using the EC-Lab software, the impedance data were fitted to an equivalent circuit that consists of a constant-phase element CPE_dl_ of the double layer in series with the parallel combination of a constant-phase element CPE_bulk_ of the bulk electrolyte and a resistance *R*_ion_ of the electrolyte (Fig. 3, inset). The ionic conductivity, *σ*, was then calculated using the equation *σ* = *L*/(*R*_ion_*S*), where (*L*) and (*S*) are the thickness and the area of the films, respectively. To be certain that the conductivities are consistent, two different set of films were prepared, *ca.* 100 μm and *ca.* 300 μm. All reported conductivity values are averages of 3 measured samples.

### 2.8 Cyclic voltammetry

Cyclic Voltammetry (CV) data were obtained using an Arbin BT2043 battery test system and a potentiostat employing Swagelok cells. The anodic stability of the composite SPEs sandwiched between a stainless steel plate and a lithium electrode was determined in a voltage between 3 V and 5.5 V *vs.* Li/Li^+^. To determine the cathodic stability, a copper electrode was used instead of a stainless steel plate, and testing was carried out between −0.5 V and 3 V *vs.* Li/Li^+^. In both cases, three scans were conducted, at a scan rate of 0.5 mV s^−1^. All measurements were acquired at 70 °C after a dwell time of 1 h.

### 2.9 Galvanostatic cycling

Cycling tests were performed employing symmetrical Li/composite-electrolyte/Li Swagelok cells using an Arbin BT2043 battery test system. All tests were conducted at 70 °C with a current density of 0.1 mA cm^−2^ and 3 hours of charge–discharge cycles. The maximum voltage response value was set to 0.5 V.

## 3 Results and discussion

Composite electrolytes of CNF-reinforced EO-*co*-EPI statistical copolymers (molar EO : EPI ratio of 84 : 16) with different LiTFSI salt concentrations *r* were prepared by solution casting using THF as common solvent and subsequent compression moulding (see Experimental section for details). The composite electrolytes were characterised by FTIR to confirm the efficient integration of both the LiTFSI salt and the CNFs into the EO-*co*-EPI matrix copolymer (Fig. S2–S4).

### 3.1 Dispersion of CNFs in EO-*co*-EPI composite electrolyte films

**Fig. 1:**
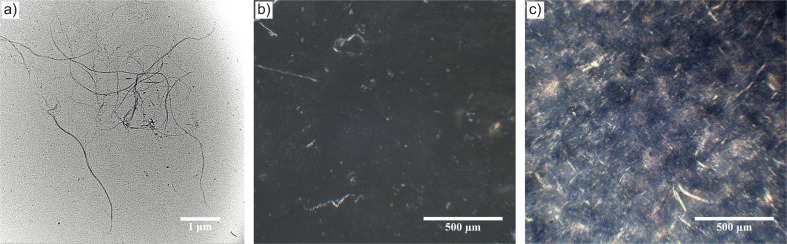
(a) Transmission electron micrograph (TEM) of CNFs. Cross-polarised optical micrographs of (b) a LiTFSI salt-doped (salt concentration *r* = 0.04) EO-*co*-EPI electrolyte film without CNFs and (c) the corresponding composite electrolyte film containing 10% w/w CNFs.

A representative TEM image of the CNFs is shown in Fig. 1a. It displays an entangled web-like structure composed of CNFs with varying lengths, making it complex to determine the CNF aspect ratios, as previously reported.^42,43^ The CNF width of 29 ± 11 nm was determined through an TEM image analysis of 150 fibres. Based on Fig. 1a, it is reasonable to assume that the CNFs have a high aspect ratio of >100.

The dispersion of the CNFs within LiTFSI salt-doped EO-*co*-EPI composite electrolyte films was visually inspected by optical microscopy in cross-polarised mode. The CNF-containing composite electrolyte films did not show any visible aggregates and appeared whitish, in contrast to films without CNFs, which were mainly translucent. Due to the different interactions of CNFs and EO-*co*-EPI statistical copolymers with polarised light, it is possible to visualise the CNF distribution inside composite electrolyte films, as shown in Fig. 1c. In the absence of CNFs, the composite electrolyte film in Fig. 1b shows no birefringence and provokes a dark micrograph when imaged between crossed polarisers, whereas the CNF-containing composite electrolyte films appear bright (Fig. 1c) due to the birefringence of the crystalline parts of the CNFs. This birefingence enables the observation of the CNF distribution and possible aggregation within the polymer matrix. The absence of larger domains in Fig. 1c suggests that large CNF aggregates are absent. Sufficient dispersion of the CNFs, as indicated in Fig. 1, suggests efficient interactions between the CNFs and the EO-*co*-EPI matrix, which was confirmed by FTIR analysis (Fig. S2†). It also allows the formation of a percolating CNF network whose ability to reinforce the polymer films^32^ was measured by dynamic mechanical analysis.

### 3.2 Dynamic mechanical analysis (DMA)

**Fig. 2:**
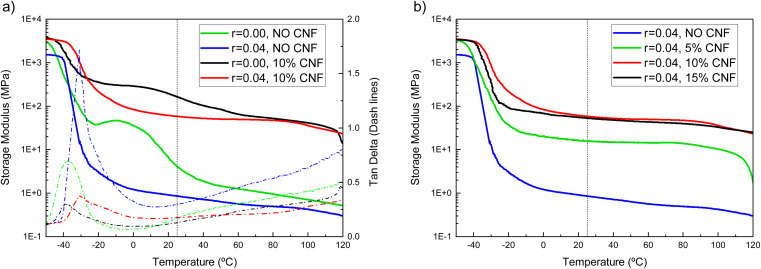
(a) DMA traces of the EO-*co*-EPI copolymer film, the LiTFSI-doped EO-*co*-EPI copolymer electrolyte film (salt concentration *r* = 0.04), the CNF-reinforced, neat EO-*co*-EPI (10% w/w CNFs, *r* = 0.00) copolymer film, and the CNF-reinforced, LiTFSI-doped EO-*co*-EPI copolymer electrolyte film (10% w/w CNFs, *r* = 0.04). Solid lines show the storage modulus (*E*′), and dashed lines represent the corresponding plot of tan *δ*, which is the ratio of the loss modulus (*E*′′) to the storage modulus. (b) DMA traces of LiTFSI-doped EO-*co*-EPI films (*r* = 0.04) with different amounts of CNFs.

The mechanical properties of the composite electrolytes were studied by dynamic mechanical analysis (DMA). Fig. 2a shows the DMA traces of the CNF-reinforced (10% w/w CNFs), LiTFSI-doped (*r* = 0.04) EO-*co*-EPI copolymer electrolyte along with those of control samples of the neat EO-*co*-EPI copolymer and the EO-*co*-EPI copolymer containing only either LiTFSI (*r* = 0.04) or CNFs (10% w/w). The storage modulus *E*′ trace of the neat EO-*co*-EPI copolymer shows a glassy regime below −45 °C in which *E*′ is on the order of 3 GPa. Above this temperature, the modulus decreases as the polymer passes the glass transition temperature (*T*_g_) of −48 °C, which is reflected by a maximum in the corresponding plot of tan *δ*, *i.e.* the ratio of the loss modulus to the storage modulus. The *E*′ trace plateaus at *ca.* 45 MPa in the temperature range between −20 °C and *ca.* 5 °C. In this regime, crystalline EO domains reinforce the rubbery amorphous domains. These EO crystals melt as the temperature increases; above *ca.* 40 °C, the *E*′ trace shows a rubbery plateau at around 1 MPa likely resulting from entanglements of the copolymer chains. The DMA data of the LiTFSI-doped EO-*co*-EPI copolymer reflect that the introduction of the LiTFSI salt causes the *T*_g_ to increase slightly to −38 °C. This increase in *T*_g_ is attributed to transient cross-links between Li ions and ether oxygens, which reduce the segmental mobility of the EO-*co*-EPI copolymer chains.^39,44–46^ Additionally, the presence of LiTFSI completely suppresses the crystallisation of the EO segments^26^ causing the onset of the rubbery regime to drop to *ca.* −10 °C. Fig. 2a also shows that the introduction of 10% w/w CNFs leads to a dramatic reinforcement of both the neat and the salt-doped EO-*co*-EPI copolymer without affecting the *T*_g_, which is consistent with previous work on similar systems.^25,27^ In the composite EO-*co*-EPI electrolytes, the ability of the EO segments to crystallise is governed by the absence or the presence of LiTFSI so that the Li-salt-free composite shows a somewhat higher storage modulus than the Li-doped composite electrolyte at temperatures below *ca.* 60 °C. Importantly, the salt-doped composite electrolyte shows a rubbery plateau that extends from *ca.* −10 °C to 120 °C in which the storage modulus assumes values between 64 and 49 MPa, which is two orders of magnitude higher than the correspondent CNF-free material (Table 1).

**Table 1:** Comparison of the storage modulus *E*′ at 25 °C, at 95 °C, and at the respective *T*_g_ of neat, CNF-reinforced and LiTFSI-doped EO-*co*-EPI copolymer films

Composition	Storage modulus *E*′ at 25 °C (MPa)	Storage modulus *E*′ at 95 °C (MPa)	*T*_g_ (°C)	Normalised tan *δ*-peak area^c^
EO-*co*-EPI	6.2 ± 0.1	0.84 ± 0.02	−38	1
EO-*co*-EPI + LiTFSI^a^	0.9 ± 0.2	0.5 ± 0.1	−31	3.52
EO-*co*-EPI + CNFs^b^	158 ± 7	47 ± 4	−38	0.79
EO-*co*-EPI + LiTFSI^a^ + CNFs^b^	64 ± 10	49 ± 12	−31	1.64

a *r* = 0.04.

b 10% w/w.

c integral over the maximum in the tan *δ* curve in Fig. 2a divided by the value of corresponding integral obtained for the neat EO-*co*-EPI copolymer sample.

Further inspection of the DMA traces shows that the integrated area of the tan *δ* peaks of the CNF-free samples is much larger than that of the CNF-containing samples, indicating changes in polymer chain dynamics. This integral is correlated with the energy dissipation in the polymer, typically in the temperature range around the glass transition temperature or a phase transition. The fact that the CNF-containing samples show a much smaller tan *δ* peak than the CNF-free reference (Table 1 the peak area normalised with respect to the peak area corresponding to neat EO-*co*-EPI) is a further indication of the increased elastic response afforded by the CNF addition, as the CNFs impose restrictions on the motion of polymer segments through fibre-matrix and fibre–fibre interactions. Consequently, less energy is dissipated during mechanical vibrations compared with CNF-free samples. Note that energy dissipation increases upon LiTFSI addition because crystallisation of the EO segments is suppressed.

Given the fibrillar nature of the CNFs, the concentration of 10% w/w used for the presently-investigated composite electrolyte should – in the absence of aggregation – lead to the formation of a percolating CNF network, which is the dominant reinforcement mechanism in nanocomposites.^32^ Therefore, further increasing the CNF content should not significantly increase the *E*′, which was confirmed by testing the mechanical properties of a CNF-reinforced composite electrolyte with different amount of CNFs. Indeed, while a reduction of the CNF content from 10% w/w to 5% w/w led to a reduction of the storage modulus, an increase to 15% w/w brought no gain in reinforcement (Fig. 2b), which is in agreement with previous studies.^40^

### 3.3 Electrochemical impedance spectroscopy (EIS)

**Fig. 3:**
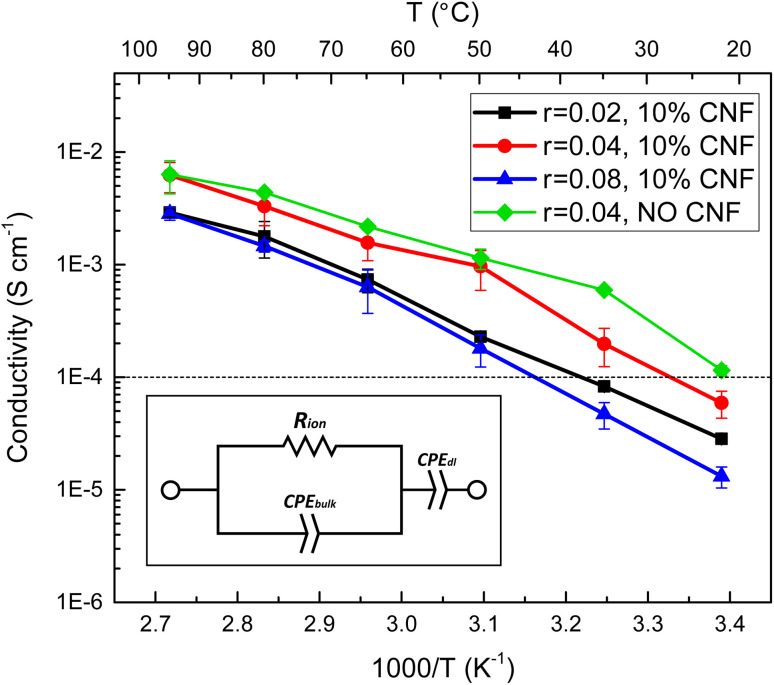
Comparison of temperature-dependent ionic conductivities for EO-*co*-EPI composite electrolyte films with varying salt concentrations, with 10% w/w CNFs and without CNF reinforcement. The conductivity was determined from the resistance obtained by fitting of impedance spectra to the equivalent circuit shown in the inset.

The relationship between salt concentration and ionic conductivity of PEO-based electrolytes is nonmonotonic due to two opposing effects.^47^ The mobility of polymer segments in the amorphous state decreases with increasing salt concentration due to the interactions between the lithium ions and the polymer segments. The concentration of Li^+^ ions, on the other hand, increases with the salt concentration, if complete dissociation is possible. The CNF-reinforced EO-*co*-EPI composite electrolyte films studied here show a similar behaviour based on EIS analysis (Fig. 3). Their ionic conductivity increases across the entire temperature range as the LiTFSI concentration is increased from *r* = 0.02 to *r* = 0.04 (molar ratio of Li^+^ ions to EO monomers), but decreases if the salt concentration is further increased to *r* = 0.08. The concentration dependence of the conductivity is less pronounced at higher temperatures where the mobility of polymer segments is higher. The relatively low *r* value is consistent with previous work on LiClO_4_-doped EO-*co*-EPI showing an optimal salt concentration around *r* = 0.033 and is likely due to the EPI units in the backbone of the copolymer, which have weaker interactions with Li^+^ than the EO units.^39^ In PEO-based SPEs, a Li^+^ ion is typically coordinated by 4 to 6 ether oxygens of PEO, the backbone of which wraps around the ion;^48,49^ the concentration of such configurations, which require closely spaced EO units, is reduced in statistical EO-*co*-EPI copolymers.

Comparing the ionic conductivity of CNF–reinforced EO-*co*-EPI and CNF-free EO-*co*-EPI electrolytes at the same LiTFSI concentration (*r* = 0.04) shows that the addition of 10% w/w CNFs does not significantly affect the conductivity, particularly at high temperatures (Fig. 3). However, EO-*co*-EPI composite electrolytes with a CNF content higher than 10% w/w exhibit much lower ionic conductivity (Fig. S7†), without any further improvement in mechanical properties (Fig. 2b), in agreement with previous work.^40^ Note that in contrast to the results by Schroers *et al.*,^40^, who studied EO-*co*-EPI-based electrolytes that were reinforced with CNCs and doped with LiClO_4_, conductivities close to 10^−2^ S cm^−1^ were attained in the absence of water, which is essential for use in a Li battery.

SPEs follow Vogel–Tammann–Fulcher (VTF) behaviour as the movement of ions is strongly coupled to the segmental motion of the polymer chains.^50^ The temperature dependence of the their ionic conductivity can therefore be written as1
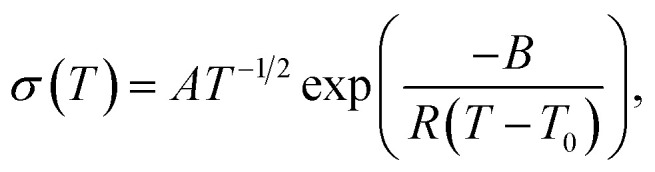
where *A* is a pre-exponential factor related to the number of charge carriers, *B* is a pseudo-activation energy, and *T*_0_ is a reference temperature, which is typically taken as 50 K below the glass transition temperature, *T*_g_, of the electrolyte.^51^ The temperature–dependent ionic conductivity shown in Fig. 3 was fit to the VTF equation in eqn (1), where *T*_0_ was fixed at 50 K below the *T*_g_ measured by DSC (Fig. S6†). The results of these fits (Table 2) show that the addition of CNFs increases the pseudo-activation energy, confirming a percolation network formed by the cellulose nanofibers that decreases the segmental mobility of the copolymer, as discussed in the previous section. However, the *A* parameter, related to the number of charge carriers, shows a significant increase when CNFs are added, which suggests that the existence of this percolation network may help increase the mobility of the ions, counteracting the detrimental effect caused in the chain mobility of the polymer.

**Table 2:** VTF parameters obtained by fitting the conductivity data shown in Fig. 3 to eqn (1) for a LiTFSI–doped EO-*co*-EPI composite electrolyte without added CNFs and LiTFSI-doped EO-*co*-EPI composite electrolytes with 10% w/w CNFs and different salt concentrations *r*

SPE	*A* (S cm^−1^ K^1/2^)	*B* (kJ mol^−1^)	*T*_0_ (K)
EO-*co*-EPI, *r* = 0.04	27 ± 4	8.3 ± 0.4	183
EO-*co*-EPI, 10% w/w CNFs, *r* = 0.04	134 ± 22	10.8 ± 0.5	183
EO-*co*-EPI, 10% w/w CNFs, *r* = 0.02	75 ± 17	11.3 ± 0.7	179
EO-*co*-EPI, 10% w/w CNFs, *r* = 0.08	104 ± 8	11.1 ± 0.2	192

Based on the performance of the different compositions of the CNF-reinforced, LiTFSI-doped EO-*co*-EPI composite electrolytes in terms of the ionic conductivity *σ* (Fig. 3 and S7†) and the storage modulus *E*′ (Fig. 2 and S5†) studied, an optimum composition of 10% w/w CNFs and an LiTFSI concentration of *r* = 0.04 was identified. The room temperature performance of EO-*co*-EPI composite electrolytes having the optimal composition is compared in Table 3 to the performance of similar composite electrolytes reported in the literature. With a value of *E*′ = 64 MPa, the storage modulus in the present work is in the upper third in terms of stiffness. The overall stiffness range is not very wide as for most of the composite electrolytes, the reinforcement was tuned to yield *E*′ values of several tens of MPa, which was shown to be sufficient for efficient suppression of Li dendrites.^28,29^ The ionic conductivity of the different systems, on the other hand, exhibits a wider range, from 10^−7^ S cm^−1^ to 10^−3^ S cm^−1^ at room temperature. The values of 6 × 10^−5^ S cm^−1^ at 25 °C and 7 × 10^−3^ S cm^−1^ at 95 °C place the present work close to the highest conductivities reported for PEO-based composite electrolytes. This is noteworthy because the present work is the only composite electrolyte with a PEO-based copolymer as the matrix instead of the PEO homopolymer used in most previous work.

**Table 3:** Comparison of this work and various other cellulose–reinforced, dry PEO-based composite polymer electrolytes from the literature in terms of the storage modulus *E*′(*T*) at *T* = 25 °C and the ionic conductivity *σ*(*T*) at *T* = 25 °C as well as *T* = 100 °C

Composition	*E*′(*T* = 25 °C) (MPa)	*σ*(*T* = 25 °C) (S cm^−1^)	*σ*(*T* = 100 °C) (S cm^−1^)	Ref.
PEO^a^ + CNCs + LiTFSI	≈60	1 × 10^−5^	5 × 10^−4^	23
PEO + CNCs + LiTFSI	195	1 × 10^−7^ at 20 °C	3 × 10^−4^	25
PEO + CNCs + LiTFSI	≈100	1 × 10^−7^	5 × 10^−4^	26
PEO + CNCs + LiTFSI	≈15	5 × 10^−6^	3 × 10^−4^ at 60 °C	24
PEO + PCA^b^ + LiBOB^c^ + cellulose nonwoven	45	1 × 10^−5^ at 20 °C	6 × 10^−4^	21
POSS-PEG^d^ + MC^e^ + LiClO_4_^f^	32	1 × 10^−5^ at 30 °C	1 × 10^−4^ at 90 °C	20
PEO + HEMC^g^ + LiTFSI	47	1 × 10^−4^ at 30 °C	2 × 10^−3^ at 60 °C	28
PEO + BC^h^ + LiTFSI	77	3 × 10^−5^	1 × 10^−3^ at 80 °C	29
PEO + CNFs^i^	≈45	2 × 10^−7^ at 30 °C	3 × 10^−4^ at 90 °C	52
PEO + CNFs + LiTFSI	n/a	3 × 10^−5^	6 × 10^−4^ at 70 °C	38
EO-*co*-EPI + CNFs + LiTFSI	64	6 × 10^−5^	7 × 10^−3^ at 95 °C	This work

a Cross-linked.

b Poly (cyano acrylate).

c Lithium bis(oxalate)borate.

d Pegylated polyoctahedral silsesquioxanes.

e Methyl cellulose.

f Lithium perchlorate.

g Methyl 2-hydroxyethyl cellulose.

h Bacterial cellulose.

i Salt not specified.

### 3.4 Electrochemistry

Electrochemical tests were carried out for the best-performing composite electrolyte in terms of ionic conductivity and mechanical properties, *i.e.* EO-*co*-EPI with *r* = 0.04 and 10% w/w CNFs. Cyclic voltammetry (CV) measurements were carried out using Li/composite-electrolyte/Cu cells in a voltage range of −0.5 V to 3 V to measure the cathodic stability while Li/composite-electrolyte/stainless-steel cells were used in a CV voltage range of 3 V to 7 V to measure the anodic stability.

**Fig. 4:**
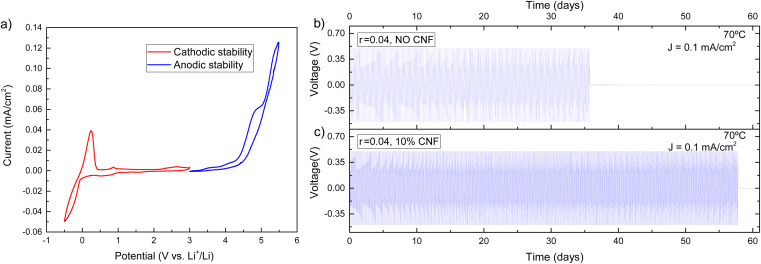
(a) Cyclic voltammogram of an EO-*co*-EPI composite electrolyte with 10% w/w CNF and an LiTFSI concentration of *r* = 0.04 over a potential range of −0.5 V – 5.5 V, at a scan rate of 0.5 mV s^−1^ and a temperature of 70 °C. (b and c) Galvanostatic cycling of an EO-*co*-EPI electrolyte with *r* = 0.04, (b) without CNFs, and (c) with 10% w/w CNFs. The tests were conducted at 70 °C with 3 hours of charge–discharge cycles at a current density of 0.1 mA cm^−2^.

The cyclic voltammogram in Fig. 4a exhibits a stability window between 0 V and 4 V, which is an essential requirement for using the EO-*co*-EPI composite electrolyte in Li batteries. In particular, the electrochemical stability window of the electrolyte (Fig. S9†) is not significantly affected by the addition of CNFs. The prominent feature in the CV curve at −0.5 V arises from a pseudo-reversible process of lithium deposition that leads to an alloy of the Cu cathode and metallic lithium.^53^ Above 4 V, oxidation of the EO-*co*-EPI copolymer sets in.

To use metallic lithium as an anode, the composite electrolyte should be able to suppress Li dendrite growth. For this purpose, the ability of the CNF-reinforced EO-*co*-EPI composite electrolyte for lithium plating and stripping was tested by performing galvanostatic cycling in a symmetric Li/composite-electrolyte/Li cell. In these experiments, the current density was fixed while the cell voltage is open (note that a voltage threshold of 0.5 V was used). Fig. 4b and c shows the voltage profiles of CNF–reinforced and CNF-free EO-*co*-EPI composite electrolytes as a function of time at a current of 0.1 mA cm^−2^ and a temperature of 70 °C. While the cell voltage drops after about 35 days for the CNF-free electrolyte, the CNF–reinforced electrolyte enabled stable operation for more than 55 days until a voltage drop was observed. Similar improvements of the cyclability were previously demonstrated in cellulose–reinforced PEO electrolytes, which was attributed to the suppression of dendrites by the added cellulose.^28,29^ The extended cycle life for the cell with a CNF-reinforced composite electrolyte is also reflected in the significantly larger amount of the total charge passed of about 500 C cm^−2^ until failure, compared to about 310 C cm^−2^ until failure of the cell using the CNF-free electrolyte (Fig. S10†). Assuming cell failure is caused by Li dendrites, the cycling results suggest that CNF reinforcement improves the resistance against dendritic growth.

## 4 Conclusion

We produced a composite polymer electrolyte comprised of a PEO-based statistical copolymer and cellulose nanofibers. This approach enables facile tuning of the ionic conductivity *σ* and storage modulus *E*′ of the composite electrolyte, delivering a competitive room-temperature performance of *σ* = 6 × 10^−5^ S cm^−1^ and *E*′ = 64 MPa. Importantly, this composite electrolyte is electrochemically stable between 0 V and 4 V, while improving cycle life of symmetrical lithium cells compared to CNF-free electrolytes, with stable operation for more than 50 days. By using a PEO-based copolymer instead of the more common PEO homopolymer matrix, this work expands the library of composite polymer electrolytes, which may enable further performance improvements in this promising class of solid electrolytes for lithium batteries.

## Conflicts of interest

The authors declare no conflicts of interest.

## Data availability

The data supporting the findings of this study are openly available at: https://doi.org/10.2303/zenodo.14041931.

## Supplementary Material

TA-011-D3TA00380A-s001

## References

[cit1] Placke T., Kloepsch R., Dühnen S., Winter M. (2017). Lithium ion, lithium metal, and alternative rechargeable battery technologies: the odyssey for high energy density. J. Solid State Electrochem..

[cit2] Lin D., Liu Y., Cui Y. (2017). Reviving the lithium metal anode for high-energy batteries. Nat. Nanotechnol..

[cit3] Horstmann B. (2021). *et al.*, Strategies towards enabling lithium metal in batteries: interphases and electrodes. Energy Environ. Sci..

[cit4] Xu W., Wang J., Ding F., Chen X., Nasybulin E., Zhang Y., Zhang J.-G. (2014). Lithium metal anodes for rechargeable batteries. Energy Environ. Sci..

[cit5] Tikekar M. D., Choudhury S., Tu Z., Archer L. A. (2016). Design principles for electrolytes and interfaces for stable lithium-metal batteries. Nat. Energy.

[cit6] Stone G. M., Mullin S. A., Teran A. A., Hallinan D. T., Minor A. M., Hexemer A., Balsara N. P. (2012). Resolution of the Modulus versus Adhesion Dilemma in Solid Polymer Electrolytes for Rechargeable Lithium Metal Batteries. J. Electrochem. Soc..

[cit7] Zheng Y., Li X., Fullerton W. R., Li C. Y. (2021). Decoupling the Modulus and Toughness Effects of Solid Polymer Electrolytes in All-Solid-State Lithium Batteries. ACS Appl. Energy Mater..

[cit8] An Y. (2022). *et al.*, Progress in Solid Polymer Electrolytes for Lithium-Ion Batteries and Beyond. Small.

[cit9] Ben youcef H., Garcia-Calvo O., Lago N., Devaraj S., Armand M. (2016). Cross-Linked Solid Polymer Electrolyte for All-Solid-State Rechargeable Lithium Batteries. Electrochim. Acta.

[cit10] Scrosati B., Vincent C. A. (2000). Polymer electrolytes: The key to lithium polymer batteries. MRS Bull..

[cit11] Quartarone E., Mustarelli P., Magistris A. (1998). PEO-based composite polymer electrolytes. Solid State Ionics.

[cit12] Jiang Y., Yan X., Ma Z., Mei P., Xiao W., You Q., Zhang Y. (2018). Development of the PEO Based Solid Polymer Electrolytes for All-Solid State Lithium Ion Batteries. Polymers.

[cit13] Berthier C., Gorecki W., Minier M., Armand M., Chabagno J., Rigaud P. (1983). Microscopic investigation of ionic conductivity in alkali metal salts-poly (ethylene oxide) adducts. Solid State Ionics.

[cit14] Xue Z., He D., Xie X. (2015). Poly(ethylene oxide)-based electrolytes for lithium-ion batteries. J. Mater. Chem. A.

[cit15] Phan T. N., Issa S., Gigmes D. (2019). Poly(ethylene oxide)-based block copolymer electrolytes for lithium metal batteries. Polym. Int..

[cit16] Zeng F., Sun Y., Hui B., Xia Y., Zou Y., Zhang X., Yang D. (2020). Three-dimensional porous alginate fiber membrane reinforced PEO-based solid polymer electrolyte for safe and high-performance lithium ion batteries. ACS Appl. Mater. Interfaces.

[cit17] Moon R. J., Martini A., Nairn J., Simonsen J., Youngblood J. (2011). Cellulose nanomaterials review: Structure, properties and nanocomposites. Chem. Soc. Rev..

[cit18] dos Santos F. A., Iulianelli G. C. V., Tavares M. I. B. (2016). The Use of Cellulose Nanofillers in Obtaining Polymer Nanocomposites: Properties, Processing, and Applications. Mater. Sci. Appl..

[cit19] Thomas S. K., Parameswaranpillai J., Krishnasamy S., Begum P. M., Nandi D., Siengchin S., George J. J., Hameed N., Salim N. V., Sienkiewicz N. (2021). A comprehensive review on cellulose, chitin, and starch as fillers in natural rubber biocomposites. Carbohydr. Polym. Technol. Appl..

[cit20] Chinnam P. R., Zhang H., Wunder S. L. (2015). Blends of pegylated polyoctahedralsilsesquioxanes (POSS-PEG) and methyl cellulose as solid polymer electrolytes for lithium batteries. Electrochim. Acta.

[cit21] Zhang J., Yue L., Hu P., Liu Z., Qin B., Zhang B., Wang Q., Ding G., Zhang C., Zhou X., Yao J., Cui G., Chen L. (2014). Taichi-inspired rigid-flexible coupling cellulose-supported solid polymer electrolyte for high-performance lithium batteries. Sci. Rep..

[cit22] Zhang J., Zhao J., Yue L., Wang Q., Chai J., Liu Z., Zhou X., Li H., Guo Y., Cui G., Chen L. (2015). Safety-Reinforced Poly(Propylene Carbonate)-Based All-Solid-State Polymer Electrolyte for Ambient-Temperature Solid Polymer Lithium Batteries. Adv. Energy Mater..

[cit23] Azizi Samir M. A. S., Alloin F., Sanchez J.-Y., Dufresne A. (2004). Cross-Linked Nanocomposite Polymer Electrolytes Reinforced with Cellulose Whiskers. Macromolecules.

[cit24] Alloin F., D'Aprea A., Kissi N. E., Dufresne A., Bossard F. (2010). Nanocomposite polymer electrolyte based on whisker or microfibrils polyoxyethylene nanocomposites. Electrochim. Acta.

[cit25] Azizi Samir M. A. S., Alloin F., Gorecki W., Sanchez J.-Y., Dufresne A. (2004). Nanocomposite polymer electrolytes based on poly (oxyethylene) and cellulose nanocrystals. J. Phys. Chem. B.

[cit26] Azizi Samir M. A., Chazeau L., Alloin F., Cavaillé J. Y., Dufresne A., Sanchez J. Y. (2005). POE-based nanocomposite polymer electrolytes reinforced with cellulose whiskers. Electrochim. Acta.

[cit27] Azizi Samir M. A. S., Mateos A. M., Alloin F., Sanchez J. Y., Dufresne A. (2004). Plasticized nanocomposite polymer electrolytes based on poly(oxyethylene) and cellulose whiskers. Electrochim. Acta.

[cit28] Wu H., Wang J., Zhao Y., Zhang X., Xu L., Liu H., Cui Y., Cui Y., Li C. (2019). A branched cellulose-reinforced composite polymer electrolyte with upgraded ionic conductivity for anode stabilized solid-state Li metal batteries. Sustainable Energy Fuels.

[cit29] Li Y., Sun Z., Liu D., Lu S., Li F., Gao G., Zhu M., Li M., Zhang Y., Bu H., Jia Z., Ding S. (2021). Bacterial Cellulose Composite Solid Polymer Electrolyte With High Tensile Strength and Lithium Dendrite Inhibition for Long Life Battery. Energy Environ. Mater..

[cit30] Aulin C., Ahola S., Josefsson P., Nishino T., Hirose Y., Österberg M., Wågberg L. (2009). Nanoscale Cellulose Films with Different Crystallinities and Mesostructures—Their Surface Properties and Interaction with Water. Langmuir.

[cit31] Iwamoto S., Kai W., Isogai A., Iwata T. (2009). Elastic modulus of single cellulose microfibrils from tunicate measured by atomic force microscopy. Biomacromolecules.

[cit32] Xu X., Liu F., Jiang L., Zhu J. Y., Haagenson D., Wiesenborn D. P. (2013). Cellulose nanocrystals vs. Cellulose nanofibrils: A comparative study on their microstructures and effects as polymer reinforcing agents. ACS Appl. Mater. Interfaces.

[cit33] Changsarn S., Mendez J. D., Shanmuganathan K., Foster E. J., Weder C., Supaphol P. (2011). Biologically Inspired Hierarchical Design of Nanocomposites Based on Poly(ethylene oxide) and Cellulose Nanofibers. Macromol. Rapid Commun..

[cit34] Kose R., Kondo T. (2013). Size effects of cellulose nanofibers for enhancing the crystallization of poly(lactic acid). J. Appl. Polym. Sci..

[cit35] Safdari F., Carreau P. J., Heuzey M. C., Kamal M. R., Sain M. M. (2017). Enhanced properties of poly(ethylene oxide)/cellulose nanofiber biocomposites. Cellulose.

[cit36] Chiappone A., Nair J. R., Gerbaldi C., Jabbour L., Bongiovanni R., Zeno E., Beneventi D., Penazzi N. (2011). Microfibrillated cellulose as reinforcement for Li-ion battery polymer electrolytes with excellent mechanical stability. J. Power Sources.

[cit37] Willgert M., Leijonmarck S., Lindbergh G., Malmström E., Johansson M. (2014). Cellulose nanofibril reinforced composite electrolytes for lithium ion battery applications. J. Mater. Chem. A.

[cit38] Qin H., Fu K., Zhang Y., Ye Y., Song M., Kuang Y., Jang S.-H., Jiang F., Cui L. (2020). Flexible nanocellulose enhanced Li+ conducting membrane for solid polymer electrolyte. Energy Storage Mater..

[cit39] Gazotti W., Spinacé M., Girotto E., De Paoli M.-A. (2000). Polymer electrolytes based on ethylene oxide–epichlorohydrin copolymers. Solid State Ionics.

[cit40] Schroers M., Kokil A., Weder C. (2004). Solid polymer electrolytes based on nanocomposites of ethylene oxide-epichlorohydrin copolymers and cellulose whiskers. J. Appl. Polym. Sci..

[cit41] Kamtsikakis A., McBride S., Zoppe J. O., Weder C. (2021). Cellulose Nanofiber Nanocomposite Pervaporation Membranes for Ethanol Recovery. ACS Appl. Nano Mater..

[cit42] Shinoda R., Saito T., Okita Y., Isogai A. (2012). Relationship between length and degree of polymerization of TEMPO-oxidized cellulose nanofibrils. Biomacromolecules.

[cit43] Li Q., Renneckar S. (2009). Molecularly thin nanoparticles from cellulose: isolation of sub-microfibrillar structures. Cellulose.

[cit44] Watanabe M., Nishimoto A. (1995). Effects of network structures and incorporated salt species on electrochemical properties of polyether-based polymer electrolytes. Solid State Ionics.

[cit45] Edman L. (2000). Ion association and ion solvation effects at the crystalline-amorphous phase transition in PEO-LiTFSI. J. Phys. Chem. B.

[cit46] Hou W. H., Chen C. Y., Wang C. C. (2004). Conductivity, DSC, and solid-state NMR studies of comb-like polymer electrolyte with a chelating functional group. Solid State Ionics.

[cit47] Hallinan D. T., Balsara N. P. (2013). Polymer Electrolytes. Annu. Rev. Mater. Res..

[cit48] Mao G., Saboungi M.-L., Price D. L., Armand M. B., Howells W. S. (2000). Structure of Liquid PEO-LiTFSI Electrolyte. Phys. Rev. Lett..

[cit49] Borodin O., Smith G. D. (2006). Mechanism of Ion Transport in Amorphous Poly(ethylene oxide)/LiTFSI from Molecular Dynamics Simulations. Macromolecules.

[cit50] Zhao Q., Stalin S., Zhao C.-Z., Archer L. A. (2020). Designing solid-state electrolytes for safe, energy-dense batteries. Nat. Rev. Mater..

[cit51] Quartarone E., Mustarelli P. (2011). Electrolytes for solid-state lithium rechargeable batteries: recent advances and perspectives. Chem. Soc. Rev..

[cit52] Samad Y. A., Asghar A., Hashaikeh R. (2012). Electrospun cellulose/PEO fiber mats as a solid polymer electrolytes for Li ion batteries. Renewable Energy.

[cit53] Goulart Silva G., Lemes N. H., Polo Da Fonseca C. N., De Paoli M. A. (1996). Solid state polymeric electrolytes based on poly(epichlorohydrin). Solid State Ionics.

