# Supplementary material for: Cellulose nanofiber-reinforced solid polymer electrolytes with high ionic conductivity for lithium batteries
Source: J Mater Chem A Mater. 2023 Apr 4;11(17):9521–9. doi: 10.1039/d3ta00380a (PMC10153659; doi:10.1039/d3ta00380a)
Supplement: TA-011-D3TA00380A-s001 [file TA-011-D3TA00380A-s001.pdf]

Electronic Supplementary Material (ESI) for Journal of Materials Chemistry A.
This journal is © The Royal Society of Chemistry 2023

Cellulose nanofiber-reinforced solid polymer electrolytes with high ionic conductivity for lithium batteries

# – Supporting Information –

Cristina Prado Martínez,[†] Preston Sutton,[†,‡] Isabella Mombrini,[†,¶] Aristotelis Kamtsikakis,[†] Worarin Meesorn,[†] Christoph Weder,[†] Ullrich Steiner,[†] and Ilja Gunkel[*,†]

†*Adolphe Merkle Institute, University of Fribourg, Fribourg, 1700, Switzerland*

‡*ARC Centre of Excellence for Electromaterials Science and Institute for Frontier Materials, Deakin University, Melbourne, 3125 Australia*

¶*Electrochemical Innovation Lab, UCL, London, U.K.*

E-mail: ilja.gunkel@unifr.ch

Sample preparation

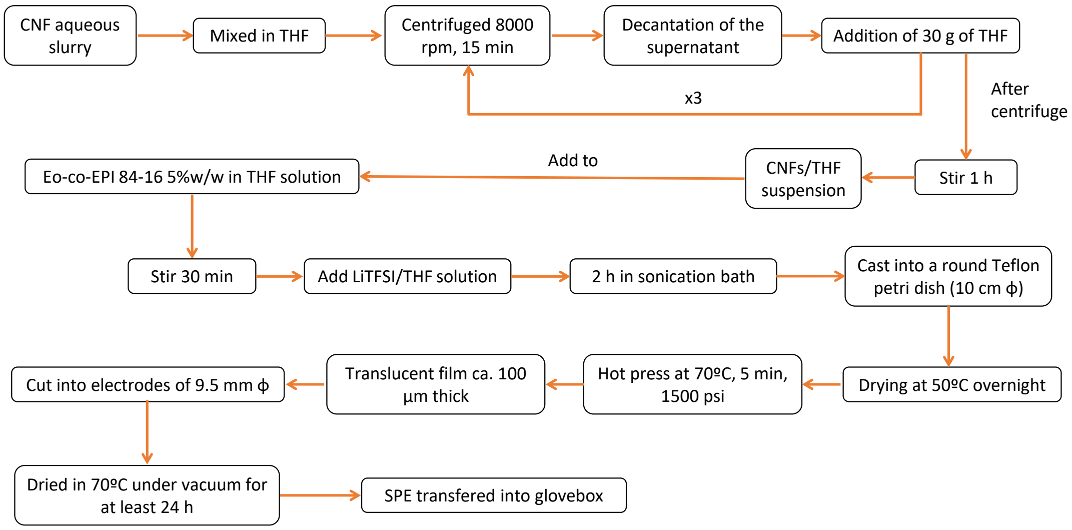

Figure S1: Flowchart for the preparation of a CNF-reinforced, LiTFSI salt-containing solid polymer electrolyte (SPE) composite using an EO–*co*–EPI statistical copolymer as the matrix.

Chemical characterisation

CNFs addition (Figure S2): Despite the overall similar spectra of the CNF-free, neat EO-*co*-EPI copolymer and the CNF-reinforced, neat EO-*co*-EPI copolymer composite, both spectra exhibit significantly reduced OH stretching bands in the region between 3500 cm^{-1} and 3000 cm^{-1} compared to the spectra of the CNFs, indicating the absence of free OH groups on the surface of the CNFs as a result of their interaction with the polymer matrix in the composite. In addition, the general systematic shift of peaks in the spectra of the EO-*co*-EPI copolymer compared to those of the CNF-containing EO-*co*-EPI composite further suggests efficient interactions between the CNFs and the matrix polymer.

LiTFSI addition (Figure S3): Analysis of the FTIR spectra of the LiTFSI salt-containing EO–*co*–EPI electrolyte compared to those of LiTFSI and EO–*co*–EPI allows evaluation of the salt-polymer interactions. For example, the observed S−N−S stretching band at around 1055 cm^{-1} in the spectra of LiTFSI and LiTFSI-doped EO–*co*–EPI clearly indicates the incorporation of the salt into the polymer matrix. In addition, the shift of the asymmetric CF_3

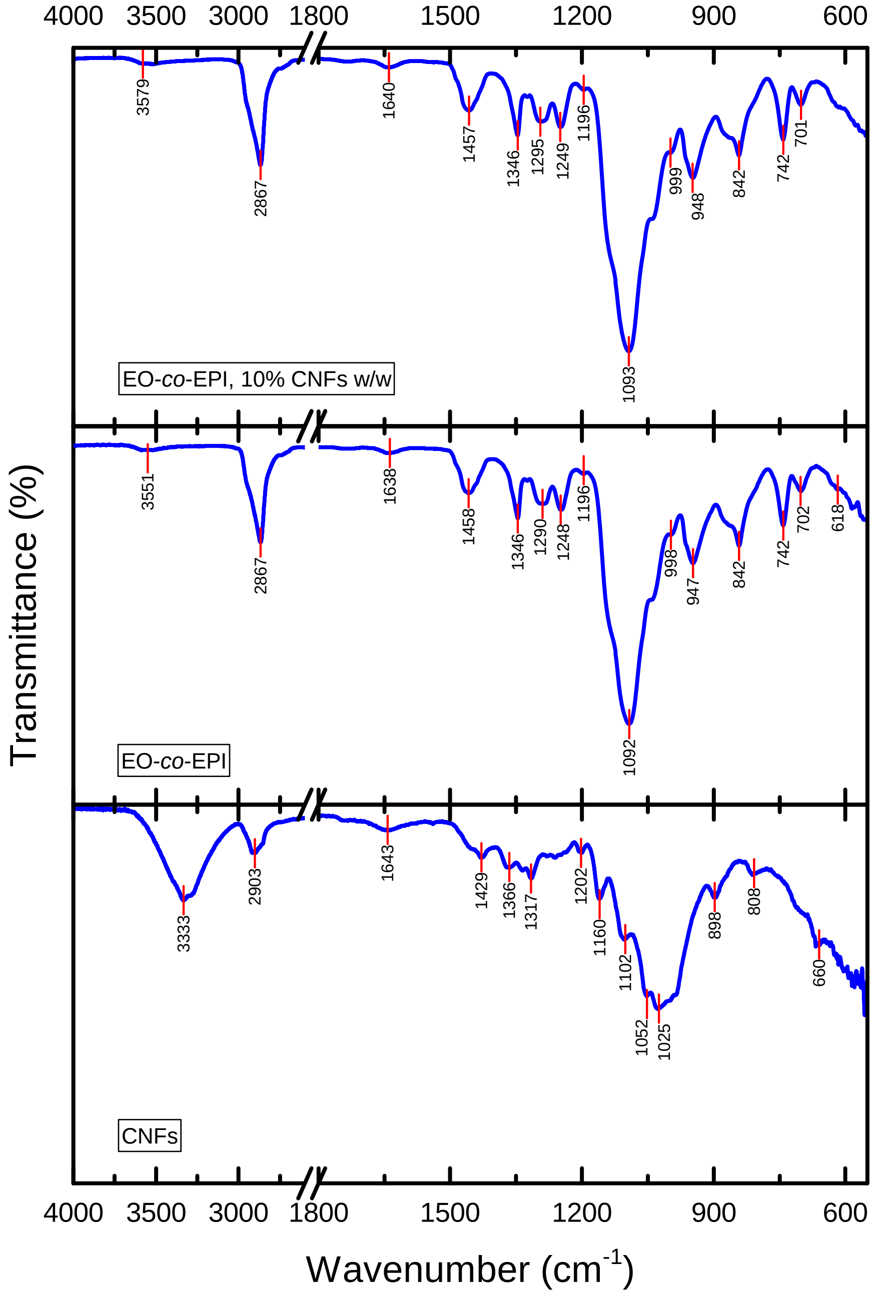

Figure S2: FTIR spectra of an EO–*co*–EPI composite reinforced with 10 % w/w CNFs and without added LiTFSI (*top*), a neat EO–*co*–EPI copolymer without added CNFs and without added LiTFSI (*middle*), and a CNF sample (*bottom*).

stretching band from 1194 cm^{-1} for LiTFSI to 1188 cm^{-1} for the LiTFSI-doped EO–*co*–EPI indicates interactions between the salt and the polymer matrix. Importantly, a similar shift observed for the C−O−C stretching band from 1092 cm^{-1} to 1089 cm^{-1} confirms the well-known complexation of the ether oxygens in EO-based polymers with Li^+ ions.[1] In addition, a shift is observed for the C−H stretching band at 2867 cm^{-1} for the neat EO–*co*–EPI to 2873 cm^{-1} band in the LiTFSI-doped EO–*co*–EPI, which also confirms polymer-salt interactions. Interestingly, while peaks are seen at 742 cm^{-1} for the neat EO–*co*–EPI characteristic of C−Cl stretching and at 746 cm^{-1} for LiTFSI attributed to S−N−S stretching, the spectra of the LiTFSI-doped EO–*co*–EPI shows a shifted peak at 740 cm^{-1}, suggesting interactions between the Cl atom of EO–*co*–EPI and LiTFSI.

Polymer composites (Figure S4): In analogy to the FTIR spectra of the LiTFSI-doped EO-*co*-EPI (Figure S3), for the CNF-reinforced, LiTFSI-doped EO-*co*-EPI (Figure S4) the S−N−S stretching band at 1056 cm^{-1} and the (shifted) C−O−C stretching band at 1090 cm^{-1} strongly confirm the successful incorporation of LiTFSI into the EO-*co*-EPI. The C−O−C stretching band appears at 1092 cm^{-1} for the neat EO-*co*-EPI, slightly shifts up to 1093 cm^{-1} for the CNF-reinforced EO-*co*-EPI (no LiTFSI), while it shifts down to 1089 cm^{-1} for the LiTFSI-doped EO-*co*-EPI (no CNF). For the CNF-reinforced, LiTFSI-doped EO-*co*-EPI, the C−O−C stretching band is at 1090 cm^{-1}, which appears to be a result of both LiTFSI and CNF addition, reflecting that the ether oxygen in C−O−C can complex Li^+ of LiTFSI, but also hydrogen bond to OH in CNFs.

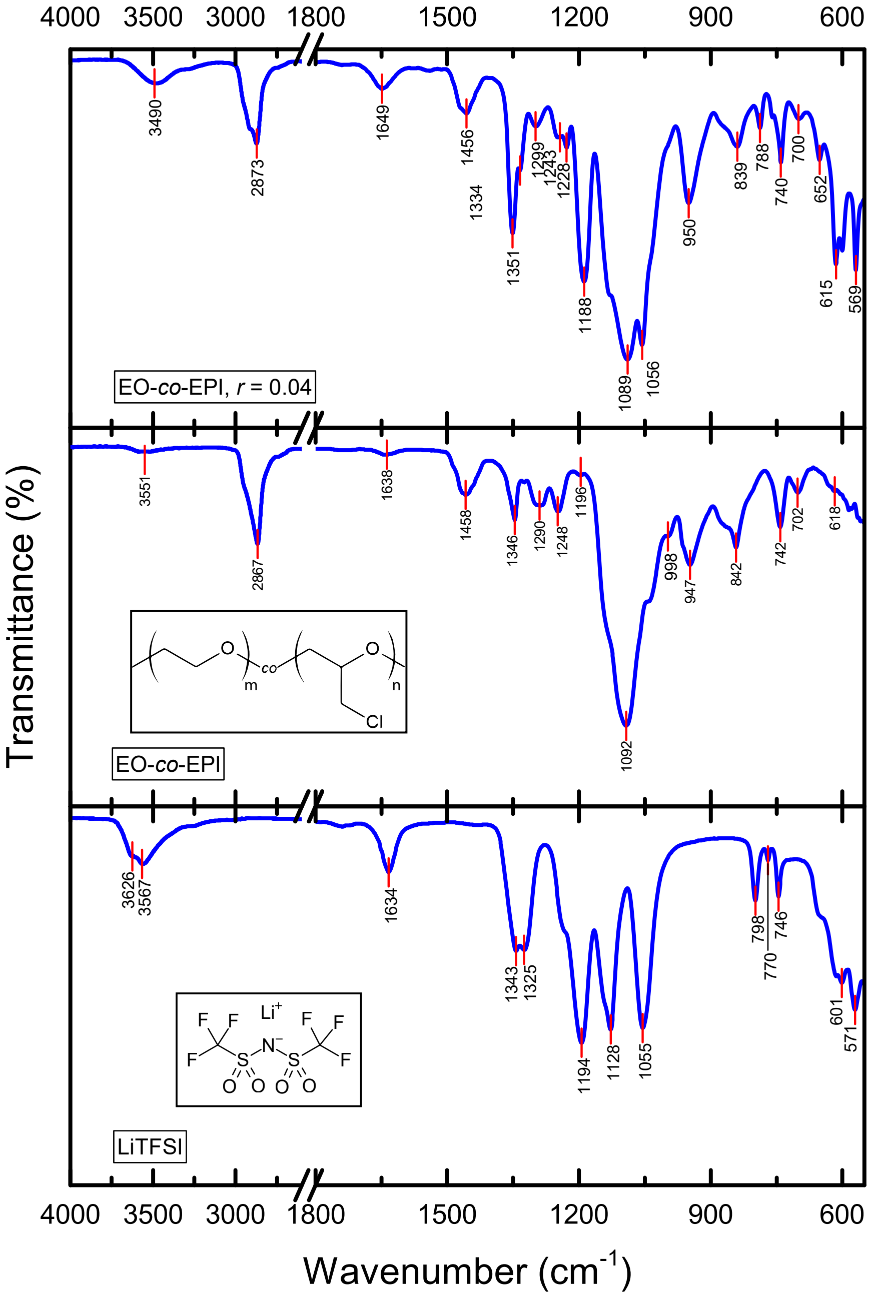

Figure S3: FTIR spectra of an EO–*co*–EPI electrolyte with an LiTFSI concentraion of $r = 0.04$ and without added CNFs (*top*), a neat EO–*co*–EPI copolymer without added LiTFSI and without CNFs (*middle*), and LiTFSI salt (*bottom*) with the chemical structures of EO–*co*–EPI and LiTFSI shown as insets.

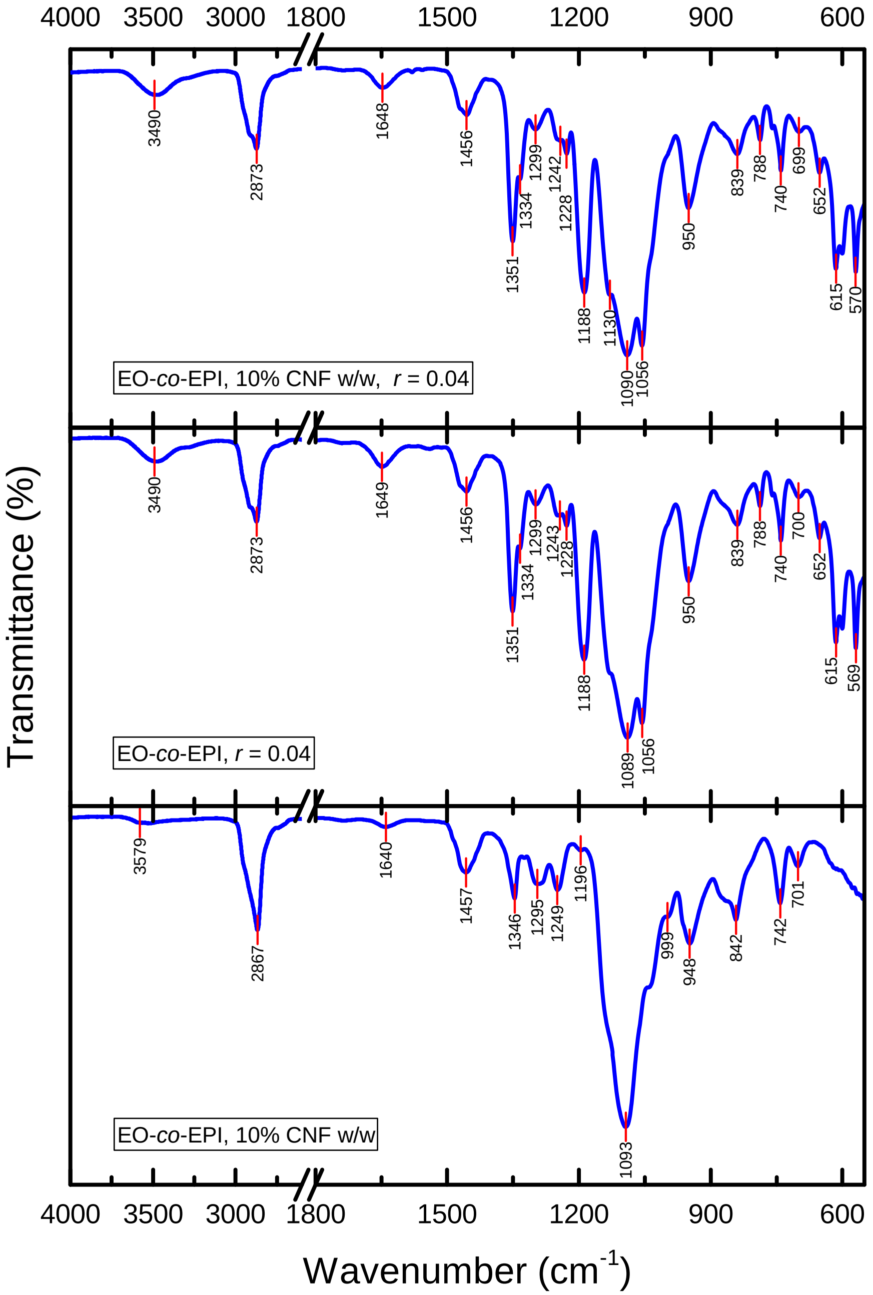

Figure S4: FTIR spectra of an EO–*co*–EPI composite electrolyte with 10 % w/w CNFs and an LiTFSI concentration $r = 0.04$ (*top*), an EO–*co*–EPI electrolyte without added CNFs and an LiTFSI concentration $r = 0.04$ (*middle*), and an EO–*co*–EPI composite with 10 % w/w CNFs and without added LiTFSI (*bottom*).

Mechanical properties

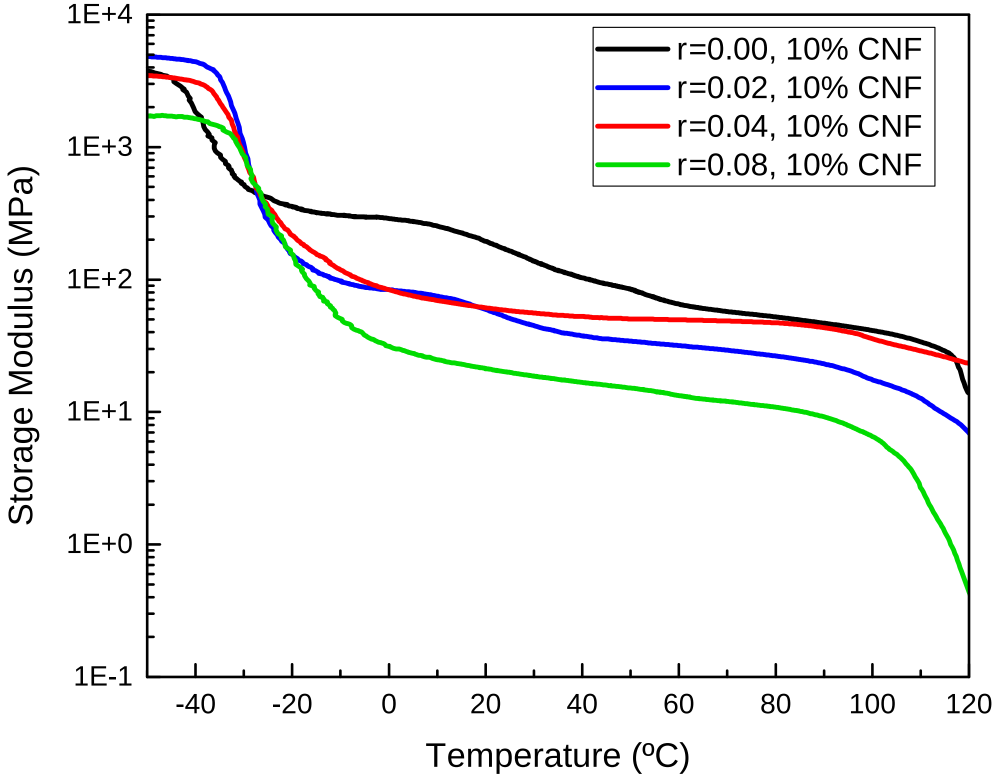

Figure S5: Storage modulus E' vs temperature of EO–*co*–EPI composite electrolytes with different LiTFSI salt concentrations ($r = 0.02$, $r = 0.04$, $r = 0.08$) and 10% w/w CNFs.

Thermal properties

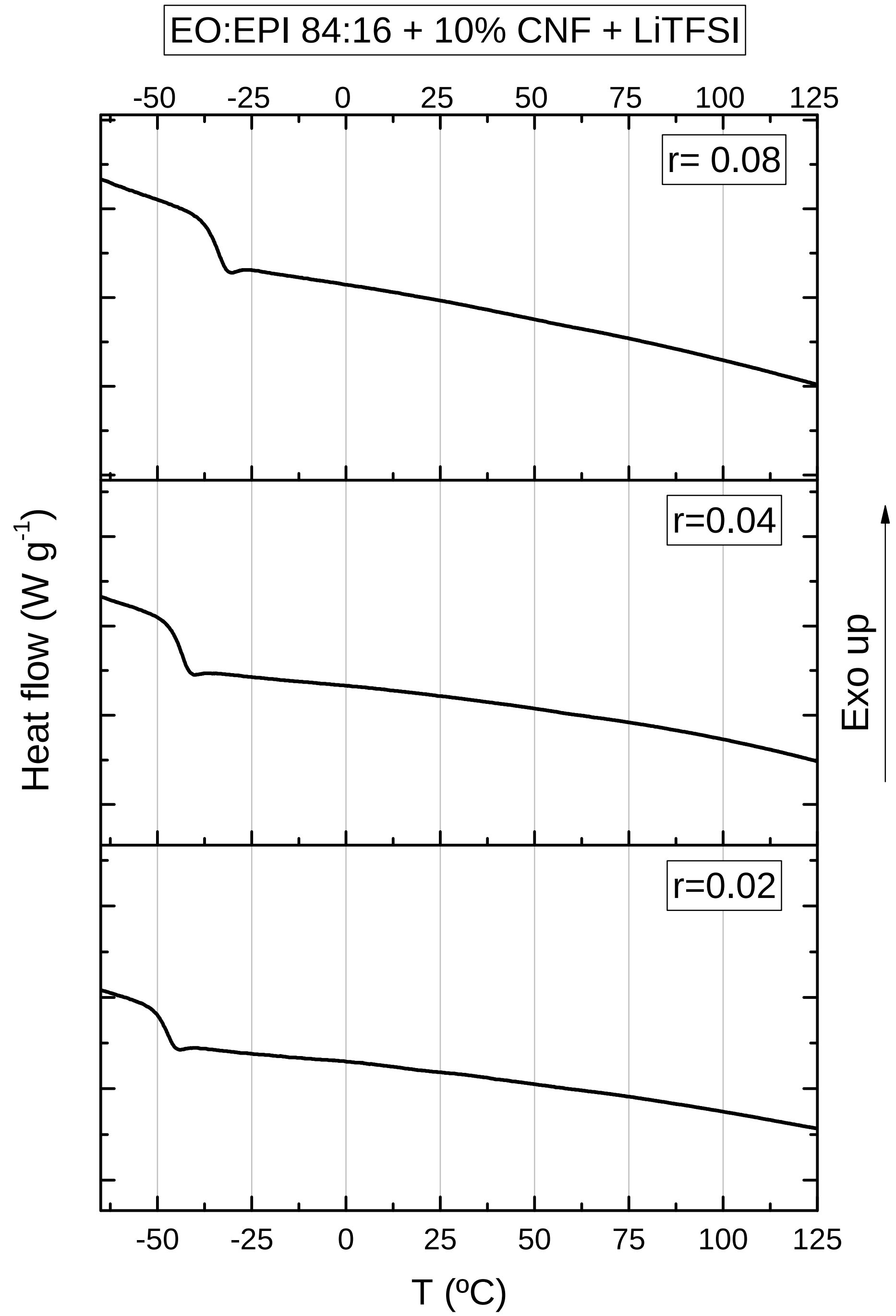

Figure S6: DSC curves of EO–*co*–EPI composite electrolytes with 10 % w/w CNFs and different LiTFSI salt concentrations r.

Characterisation of ionic conductivity

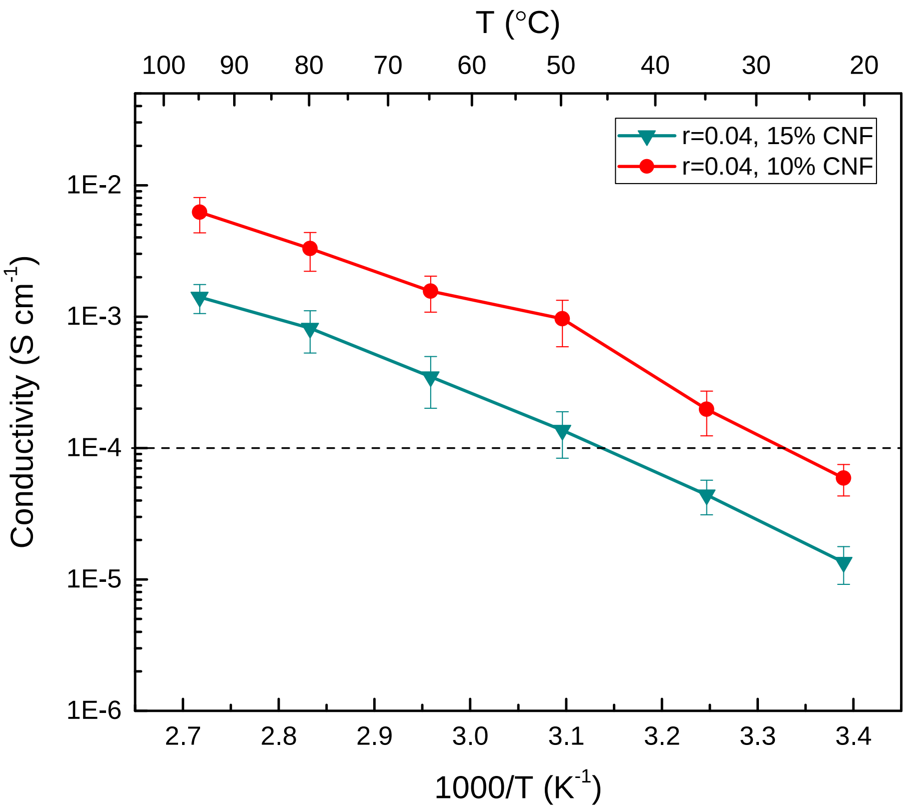

Figure S7: Comparison of the temperature–dependent ionic conductivities of the EO–*co*–EPI composite electrolytes reinforced with 10% w/w and 15% w/w CNFs and constant LiTFSI salt concentration of $r = 0.04$.

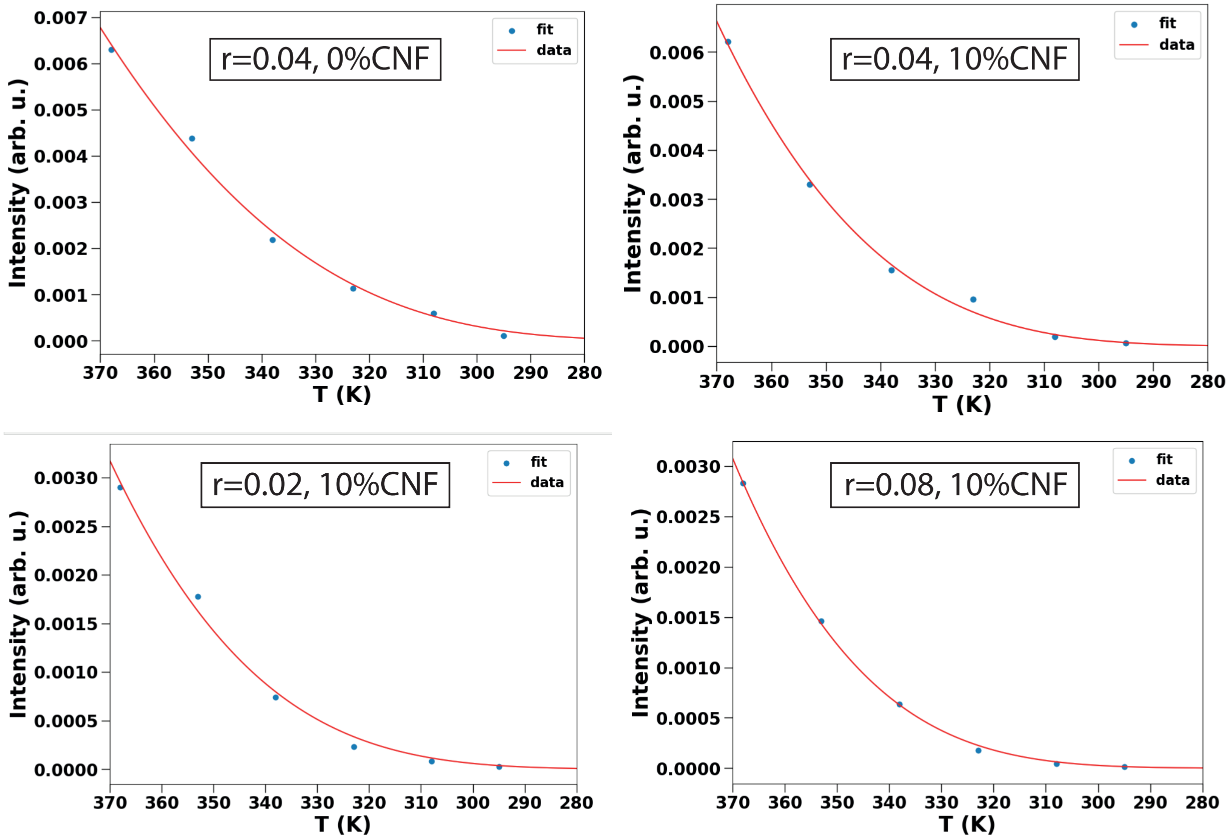

Figure S8: VTF fitting of the ionic conductivity of an LiTFSI–doped EO–*co*–EPI composite electrolyte without added CNFs as well as LiTFSI–doped EO–*co*–EPI composite electrolytes with 10% w/w CNFs and different salt concentrations r.

Electrochemical characterisation

The CV curve of an EO-*co*–EPI electrolyte with an LiTFSI concentration of $r = 0.04$ and without CNFs (Figure S9) agrees with previous work on similar systems.[2,3] For the cathodic sweep, the peak at -0.5 V is characteristic of the Li deposition in Cu, while for the anodic scan, the small peak slightly below 4 V is due to the oxidation of PEO.[4,5]

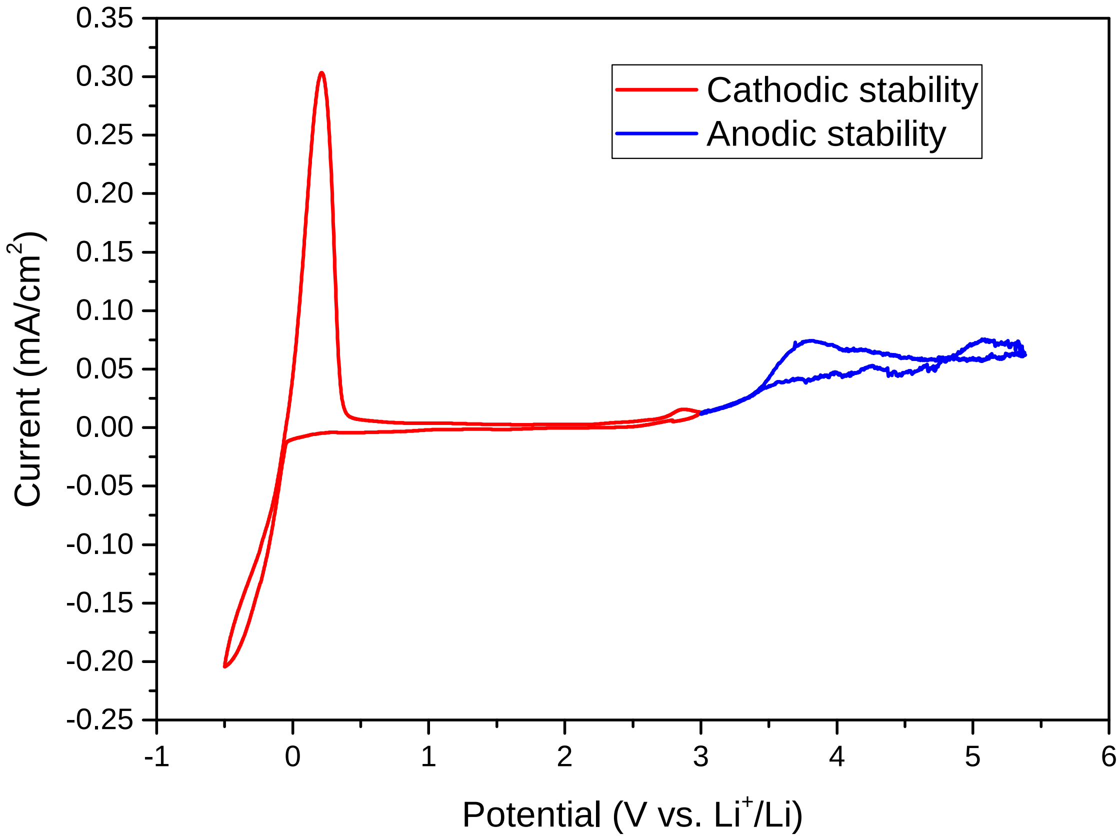

Figure S9: Cyclic voltammogram of an EO–*co*–EPI electrolyte with an LiTFSI concentration of $r = 0.04$ and without CNFs over a potential range of $-0.5\,\mathrm{V} - 5.5\,\mathrm{V}$, at a scan rate of $0.5\,\mathrm{mV\ s^{-1}}$ and a temperature of $70\,°\mathrm{C}$.

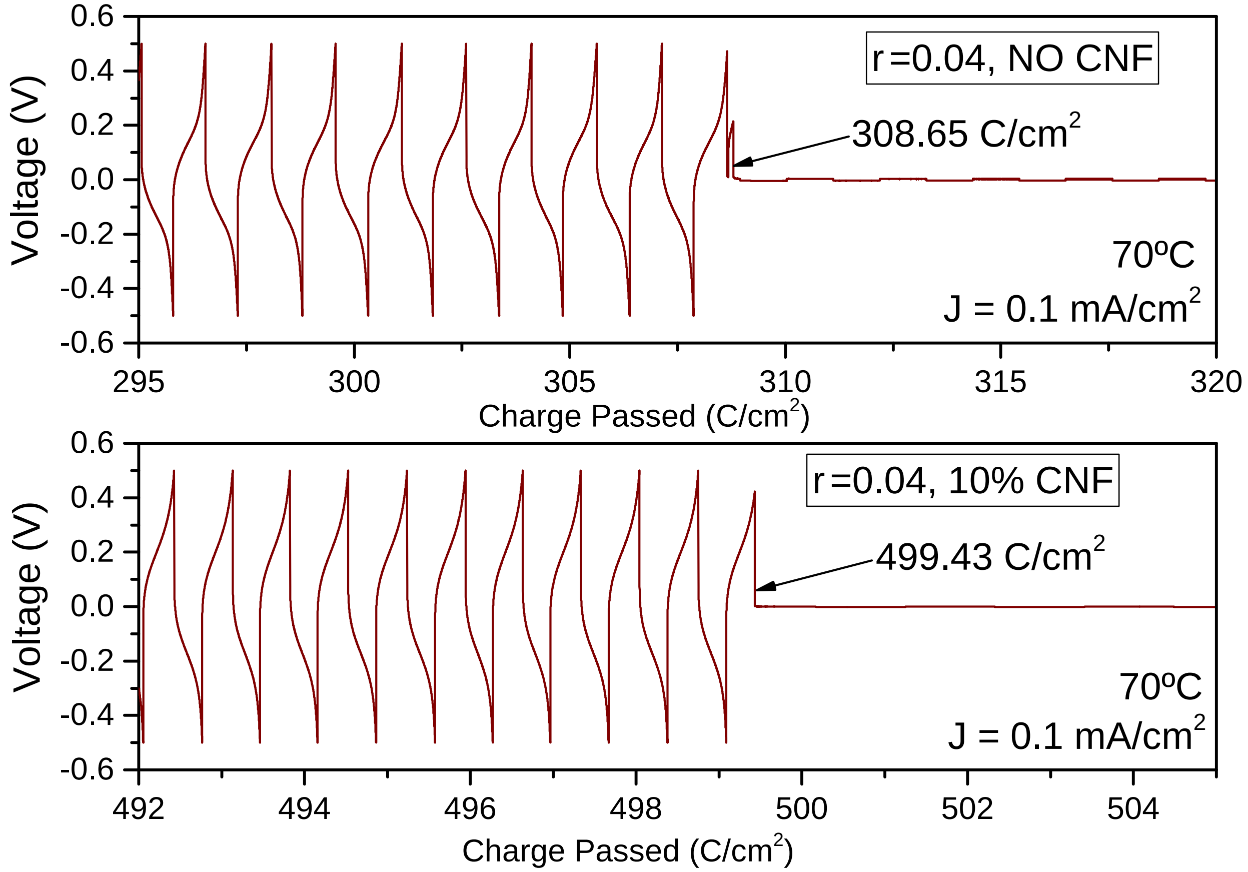

Figure S10: Plots of the voltage versus total charge passed for symmetrical Li/Li using LiTFSI–doped EO–*co*–EPI composite electrolytes with a salt concentration of $r = 0.04$ a) without CNF reinforcement, and b) with 10% w/w CNFs. In both cases the last cycles before failure are shown.

References

(1) Rey, I.; Lassègues, J.; Grondin, J.; Servant, L. Infrared and Raman study of the PEO-LiTFSI polymer electrolyte. *Electrochimica Acta* **1998**, *43*, 1505–1510.

(2) Gazotti, W.; Spinacé, M.; Girotto, E.; De Paoli, M.-A. Polymer electrolytes based on ethylene oxide–epichlorohydrin copolymers. *Solid State Ionics* **2000**, *130*, 281–291.

(3) Goulart Silva, G.; Lemes, N. H.; Polo Da Fonseca, C. N.; De Paoli, M. A. Solid state polymeric electrolytes based on poly(epichlorohydrin). *Solid State Ionics* **1996**, *93*, 105–116.

(4) Méry, A.; Rousselot, S.; Lepage, D.; Dollé, M. A Critical Review for an Accurate Electrochemical Stability Window Measurement of Solid Polymer and Composite Electrolytes. *Materials 2021, Vol. 14, Page 3840* **2021**, *14*, 3840.

(5) Xue, Z.; He, D.; Xie, X. Poly(ethylene oxide)-based electrolytes for lithium-ion batteries. *J. Mater. Chem. A* **2015**, *3*, 19218–19253.
